# The Effects of Nutritional Juice Supplementation on the Extent of Climacteric Symptoms: An Observational Study

**DOI:** 10.1155/2016/2636542

**Published:** 2016-09-07

**Authors:** Stefanie I. Siebler, Ursula Gresser, Barbara M. Richartz

**Affiliations:** ^1^Medical Office, 90402 Nuremberg, Germany; ^2^University of Munich, Munich, Germany

## Abstract

*Objective.* This study aims to evaluate the effect of daily dietary nutritional supplement on somatic, psychological, and urogenital symptoms in postmenopausal women.* Material and Methods.* In this study 28 healthy, symptomatic, peri- and postmenopausal women of 47–67 years of age were allocated to use the nutritional supplement JuicePLUS+®. Primary research parameters: Menopause Rating Scale (MRS) was used to assess menopausal symptoms at baseline and after 8 and 16 weeks of treatment. Secondary parameters: proliferation behaviour of vaginal smear was scored at baseline and after treatment.* Results.* Treatment with the supplement resulted in a reduction of somatic, psychological, and urogenital symptoms. The overall MRS score showed an average improvement of 44.01%. Most benefits were observed for the psychological symptoms irritability (60.55%) and physical and mental exhaustion (49.08%); modest effects were observed for hot flashes (44.86%) and sleeping problems (35.56%). There was a minor improvement in sexual problems; 6 women reported an increased libido. No statistically significant effect was found in vaginal dryness and proliferation behaviour of vaginal mucosa. No adverse effects were observed.* Conclusion.* Dietary nutritional supplement may constitute an effective alternative therapy to conventional alternative medicine for somatic, psychological, and sexual symptoms.

## 1. Introduction

Midlife and older women are often affected with climacteric symptoms like hot flashes, sleeping disturbance, psychological symptoms, and vaginal dryness. Vasomotor symptoms concern more than 50% of the menopausal women from premenopause to peri- and postmenopause [1] and are most strongly associated with menopausal status [2].

The Study of Women's Health Across the Nation (SWAN) from 2013 shows that the median age at the final menstrual period was 52,5 years without any difference in ethnic groups. Socioeconomic factors such as higher educational level and being employed, prior oral contraceptive use, and a better health-conscious behaviour with less alcohol and nonsmoking were associated with later age at menopause [3]. Menopausal symptoms were also influenced by demographic variables (education, employment status), lifestyle variables (smoking, activity level, and BMI), and mental healthiness [4].

Hormone supplement therapy is well established for reversing the manifestations of low estrogen levels and can reduce the frequency of hot flashes. A review from 2004 shows a significant reduction in the hot flash frequency for hormone therapy compared to placebo [5]. However, studies report side effects [5] and risks of hormone therapy, such as cardiovascular diseases and breast cancer [6, 7]. Women who cannot take hormones because of breast cancer or other risks or women who want to prevent risks of a hormone therapy search for alternative medicine for the treatment of menopausal symptoms. More than 50% of symptomatic German women with a mean age of 52.6 years have used some form of alternative medicine [8]. Available, for example, are* Cimicifuga racemosa*, red clover, or soy isoflavone extracts. The results of studies are controversial. Trials do not support the efficacy or have mixed results for the efficacy of isoflavones [9]. Cimicifuga provides evidence for the efficacy in treatment of climacteric symptoms [10].

Lifestyle variables such as nonsmoking, less alcohol, higher activity level, and lower BMI can influence menopausal symptoms [4]. For a health-conscious behaviour nutrition is of growing importance. In contrast to the desire for well-balanced nutrition there is insufficient time for preparing fresh fruit and vegetable. An enormous variety of nutrition supplements suggest a benefit for the well-being, probably also for the intensity of menopausal symptoms. We know that nutrition with nutritional supplement is important for many processes in the metabolism.

In this study we respond to the question of how far a dietary supplementation of plant substances, in this case JuicePLUS+ by The JuicePLUS+ Company, can reduce somatic, psychological, and urogenital symptoms in postmenopausal women. A publication from 2013 presented the data basis of JuicePLUS+ available to date and reported beneficial effects and side effects of the nutritional supplement [11]. Until now, data on the effect of JuicePLUS+ on menopausal symptoms have not been available.

## 2. Methods

### 2.1. Study Design and Participants

Twenty-eight women of 47–67 years of age were recruited from the medical office of gynecology situated in the center of Nuremberg to participate in an open, 16-week trial without control, designed to examine the effects of dietary supplementation of plant substances on menopausal symptoms. All patients gave consent to participate in the study.

Inclusion criteria were as follows: women were eligible for the study if they were in menopausal transition or postmenopausal, in generally good health, reporting climacteric symptoms according to the Menopause Rating Scale (MRS), and currently not using any type of systemic hormone therapy or hormonal contraceptives. Exclusion criteria were as follows: women unwilling to take daily dietary nutritional supplement for the 16-week study period, use of other food supplements, allergies to the constituents of the dietary supplementation, and current severe illness like cancer.

### 2.2. Nutritional Supplement

To evaluate the effect of daily dietary supplementation with plant substances, we selected the plant concentrate JuicePLUS+ by “The JuicePLUS+ Company Europe Ltd.”

JuicePLUS+ is an encapsulated powder concentrate made of powdered juice of 30 sorts of fruits, vegetables, and berries. There are three different powder compositions: fruit blend, vegetable blend, and berry blend. The preparation is entirely natural. It is free from gluten, sugar, lactose, artificial aromas, and colors and free from chemical stabilisers. The fructose level is unknown. Vitamin E, ascorbic acid, folic acid, and ß-carotene are admixed through the company to provide firm concentrations; the additives are of natural origin [12]. The single constituents are listed in a publication from 2013 [11].

Studies about the effect of JuicePLUS+ demonstrated numerous positive effects on the organism. Vitamins, minerals, and secondary plant substances have an impact on the immune system. JuicePLUS+ reduces the homocysteine as a marker of oxidative stress [13–15] and increases the blood level of antioxidants [16, 17]. A study from Smith et al. shows an improvement of the general condition and life quality under daily supplementation with JuicePLUS+ [18]. Another trial about the effect of JuicePLUS+ verifies a positive influence of the plant substances on the extent of treatment of resistant chronic periodontitis and a positive sense of well-being [19].

### 2.3. Treatment and Measurements

All study participants took the plant concentrate JuicePLUS+ in accordance with the manufacturer's recommendations: 2 capsules daily of the fruit, vegetable, and berry mixture.

Prior to the start of the study, the participants had undergone a standardized questionnaire on demographic characteristics, including age, educational level, age at menopause, time since menopause, and use of medication.

Data were collected at baseline and after 8 and 16 weeks of use of the nutritional supplement: In a general examination blood pressure, weight, and height were measured and the body mass index was calculated as weight (kg)/height (m^2^). The Menopause Rating Scale was used to evaluate menopause symptoms.

The MRS is composed of 11 items assessing menopause symptoms and is divided into three subgroups [20]:

1. Somatic symptoms: hot flashes, heart discomfort, sleeping problems, and muscle and joint problems.
2. Psychological symptoms: depressive mood, irritability, anxiety, and physical and mental exhaustion.
3. Urogenital symptoms: sexual problems, bladder problems, and vaginal dryness.

Each item is graded by the participant, with scores from 0 to 4 (0: absent; 1: mild; 2: moderate; 3: severe; 4: very severe). A maximal MRS score of 16 can be achieved in somatic and psychological symptoms and 12 in urogenital symptoms.

In a gynecological examination, vaginal smear was obtained from the vagina. The proliferation behaviour was scored under a light microscope two times at baseline and after 16 weeks and was classified into different categories [21].

The proliferation behaviour is an indicator for the quality of the vaginal mucosa and indirectly for the hormonal status and the level of dryness. Postmenopausal women usually show a lower proliferation behaviour and are often seriously affected by vaginal dryness.

In the absence of a placebo group in our trial, we searched for a placebo group from a comparable study about alternative medicine in treatment of menopausal symptoms. We found the double-blind, placebo controlled trial from Carmignani from 2010. He compared the effects of dietary soy supplementation, low-dose hormone therapy, and placebo on menopausal symptoms [22]. The inclusion criteria are comparable to our trial. The placebo group and the hormone therapy group consist of 20 menopausal women, between 40 and 60 years of age, who had their last menstrual period more than 12 months previously. The study design was equivalent to using Menopause Rating Scale to evaluate menopausal symptoms at baseline and after 8 (visit 2) and 16 weeks (visit 3).

### 2.4. Statistical Analysis

In our trial some women were yet in menopausal transition (last menstrual period dates back to less than 12 months). For comparison with the placebo group from [22], in which all women were postmenopausal and without hysterectomy, we divided the participants into 2 groups:

(i) Analysis group 1: all compliant women, *n* = 28.
(ii) Analysis group 2: all postmenopausal women without hysterectomy, *n* = 13, in comparison with placebo group and hormone therapy group.

Data were analyzed in both groups. Continuous data referring to women's epidemiological and clinical characteristics were analyzed descriptively, such as age, blood pressure, BMI, age at menopause, and age at the beginning of symptoms. The focus was on mean and standard deviation. The longitudinal data from Menopause Rating Scale were analyzed using median, standard deviation, and mean, and interquartile range (IQR) and 5th and 95th percentile were calculated for baseline and after 8 and 16 weeks. Changes in menopausal symptoms were addressed by absolute differences in MRS comparing visit 3 with baseline and analyzed by the Wilcoxon signed-rank test. Comparisons to results from the literature with respect to different treatment groups were done by corresponding 95% confidence intervals. As the results in [22] only show point estimates of the mean without confidence intervals, we only conclude a tendency. If the mean lies in the confidence interval (CI), no difference can be shown. If the mean lies above or below the CI, we assume a tendency at the expense or in favour of the respective group.

*P* values less than 0.05 were considered statistically significant. For analyzing the data the statistical package SAS 9.4® was used.

## 3. Results

In total, 30 women were included in the study. Compliance was high, 28 women completed 16 weeks, and only 2 women were terminated early due to relocation and were not included in the data analysis.

Table 1 shows the baseline characteristics of the participants according to the analysis group. The following results refer to analysis group 1 with all 28 participants. The mean age of the women was 54.9 years (SD 5.2). Women had been postmenopausal for a mean of 6.8 years (SD 7.7) and mean age at menopause was 48.8 years (SD 5.4).

The beginning of the first menopause symptoms was 2.4 years before starting the trial. At baseline the mean BMI was 25.2 kg/m^2^ and mean blood pressure was 126.6/78.7 mmHg. We proved no essential changes in BMI and blood pressure until visit 3.

A statistically significant decrease in the overall baseline MRS score and in the scores for all subscales was found after 16 weeks in all groups. The overall MRS score at the beginning was 17.86 at baseline and 10.00 at visit 3 in analysis group 1. This means an average improvement of 44.01%.

**Figure 1:**
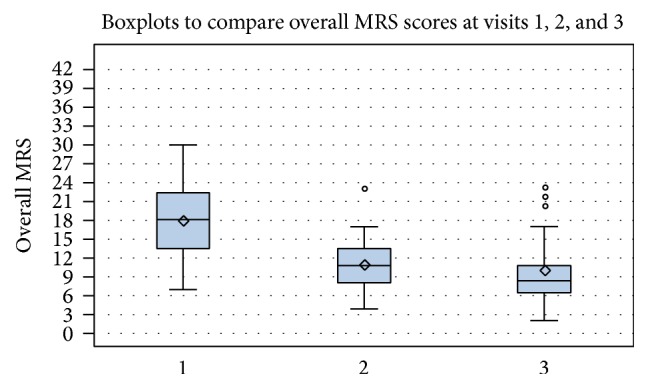
Boxplots comparing overall MRS scores at baseline and at visits 2 and 3, analysis group 1, *n* = 28. This figure reflects the course of the overall MRS score at visits 1, 2, and 3. The box and whisker plots include the median, the mean, the interquartile range (IQR: defining the inner 50% of the data), the 5th and 95th percentile (upper and lower whiskers), and outlier. ⋄, mean.

**Figure 2:**
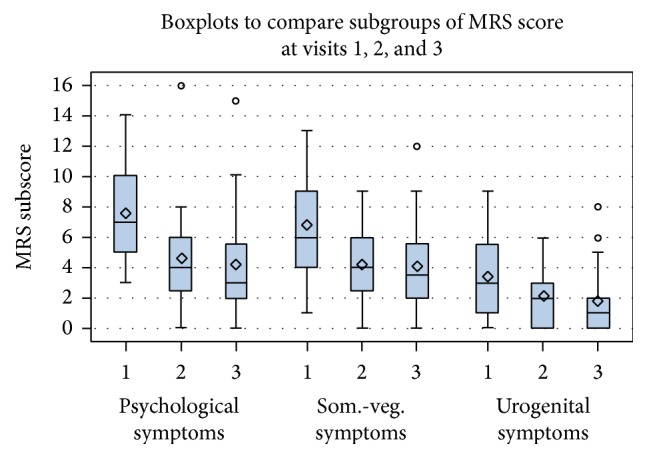
Boxplots comparing MRS subgroups at baseline and at visits 2 and 3, analysis group 1, *n* = 28. This figure reflects the course of the subgroups of MRS score at visits 1, 2, and 3. The box and whisker plots include the median, the mean, the interquartile range (IQR: defining the inner 50% of the data), the 5th and 95th percentile (upper and lower whiskers), and outlier. ⋄, mean.

The boxplots in Figures 1 and 2 reflect the course of the overall MRS and the MRS score in the subgroups at baseline and at visits 2 and 3 in analysis group 1 with all women (*n* = 28).

The highest MRS score at baseline was in the psychological symptoms group with 7.61 points. Intergroup analysis revealed the best improvement in psychological symptoms with an average of 45.07% (−3,43). The reduction of the MRS score referring to irritability was 60.55% and physical and mental exhaustion was 49.08%.

A modest effect with 40.32% (−2.75) was observed for somatic symptoms. Hot flashes and sleeping problems decreased by 44.86% and 35.56%. Minor improvement was observed concerning urogenital symptoms; we found a reduction of overall minus 1.68 points.  Table 2 reflects the differences in MRS score between the subgroups' psychological, somatic, and urogenital symptoms at visit 3 and baseline.

In the group of urogenital symptoms, sexual problems (−0.79) improved most likely; 6 women reported an increased libido (21.43%). Bladder problems showed a minus of 0.5 points.

There was no statistically significant positive effect in vaginal dryness and proliferation behaviour of vaginal mucosa after daily supplement of the nutritional supplement. The score in vaginal dryness improved by minus 0,39 scores.

Table 3 reflects the change in proliferation behaviour in a contingency table. At the beginning of trial 9, 28 women were in category 1 (Schmitt's proliferation value of 1 and 1-2) which means a very low hormonal level of the vaginal mucosa. Two women were in category 2 (proliferation value of 2 and 2-3) and 17 in category 3 (proliferation value of 3 and 3-4). Many women stayed in their category, 6 in category 1 and 17 in category 3 at visits 1 and 3, which means that there appeared to be no statistically important changes for the proliferation behaviour of vaginal mucosa.

The best improvement over time was found already after 8 weeks. The continuing daily intake of the supplement only leads to a marginal improvement of the overall MRS score for the last 8 weeks. No adverse effects were observed.

There was no significant difference in overall MRS score and in subscales comparing the subgroups of all women (*n* = 28) and all postmenopausal women without hysterectomy (*n* = 13).

For comparing the effects with a placebo and a hormone therapy group, we used the results from the trial of Carmignani [22].

Table 4 reflects that the differences in the mean values of the placebo group in all subgroups and in the overall MRS score are contained in the corresponding 95% confidence intervals of analysis group 2. This leads to the conclusion that there is no statistically significant difference in the results of both analysis groups.

There is no significant difference between the effects of the nutritional supplement and placebo. Comparing our results with the hormone therapy group we observed no significantly better effect of systemic hormone therapy in the treatment of psychologic and urogenital symptoms as well. In contrast, looking at the differences in the mean values of the hormone therapy group, there is a trend in an improved efficacy of hormone therapy.

The supplement with JuicePLUS+ is at least obtaining similar effects on menopausal symptoms like placebo. JuicePLUS+ is an effective and reasonable alternative to other conventional alternative medicine without side effects.

## 4. Discussion

This is the first trial to investigate the efficacy of daily dietary supplementation with encapsulated powder concentrate made of powdered juice for improving menopausal symptoms using Menopause Rating Scale and validating measures of proliferation behaviour of vaginal cells. In this trial, treatment with the concentrated nutritional supplement JuicePLUS+ improved overall menopausal symptoms from baseline to 16 weeks. The highest reductions were found in psychological symptoms irritability and physical and mental exhaustion. Modest effects were observed for the single symptoms hot flashes and sleeping problems. Minor reduction was in urogenital symptoms. There was no significant effect in vaginal dryness and proliferation behaviour of vaginal mucosa.

The mean age at menopause was 48.8 years (SD 5.4). This is similar to results of other trials [3, 23].

### 4.1. Somatic Symptoms

In this trial, hot flashes and sleeping disturbances are afflicting symptoms for all women. This is consistent with results from a trial of 2014 [8]. The single items hot flashes and sleeping disturbances improved significantly; a slightly minor improvement was found for heart discomfort. In the literature, we found inconsistent effects of other nutritional supplements. Trials about the effects of black cohosh and isoflavones made of soy or red clover showed positive effects on somatic symptoms [24–26]. Despite these trials, a review from 2006 reported mixed effects of soy extracts and no reduction of hot flashes with red clover [9].

### 4.2. Psychological Symptoms

Some of the most common and bothersome menopausal symptoms are depressive mood, irritability, anxiety, and physical and mental exhaustion. A trial from 2015 showed a strong association between menopausal symptoms and psychiatric diseases [27]. All psychologic symptoms improved significantly in our trial, more than somatic and urogenital symptoms. The intake of the plant concentrate was associated with an increased feeling of well-being. Comparable effects have also been described in a recent study [19]. Similar but less positive trend was found in the trial of Carmignani [22]. A study about black cohosh and St. John's wort showed a statistically significant improvement of depressive mood [28]. Also red clover may reduce psychologic symptoms [26].

Our finding differs from results published in the literature, in which no improvement is seen in psychological symptoms with neither plant substances nor hormone therapy [29]. A review from 2015 about the effect of hormone therapy on depressive mood found that estrogen improved psychological symptoms but a combined hormone therapy has a negative effect [30]. Therefore, hormone therapy is not indicated for treatment of psychological symptoms. For this reason supplementation with plant concentrate might be an alternative in the treatment of depressive mood, irritability, anxiety, and physical and mental exhaustion.

### 4.3. Urogenital Symptoms

A well functioning sexuality is fundamental for menopausal women. The Study of Women's Health Across the Nation came to the result that most women, between 42 and 52 years of age, describe sexuality as being still important to very important for them [31]. Unfortunately vaginal pain and dryness increase in the late menopause and the desire for sexuality and sexual activity decreases [32]. The cause of urogenital problems is the decrease of estrogen following vaginal atrophy and sexual dysfunction. The increasing prevalence of vaginal atrophy is most between the early and late menopause [33].

Our trial reflected a modest reduction in urogenital symptoms. Most likely, there was an improvement in sexual problems. Some women reported increasing libido. In the trial of Carmignani, there was no change in sexual symptoms neither with isoflavone nor with hormone therapy [34]. Despite other studies showing evidence to the contrary [26, 35], in this trial vaginal dryness showed hardly any improvement. All participants reported a modest reduction in intensity of bladder problems under supplementation with the nutritional supplement.

There was no statistically significant positive effect in vaginal dryness and proliferation behaviour of vaginal mucosa after daily supplement of nutritional supplement. Vaginal cytology showed no improvement in quality of the vaginal mucosa. This is opposite to the results of the trial of Carmignani [34].

### 4.4. Comparing with Placebo and Hormone Therapy

One important question to answer is how far the placebo effect influenced the result of this trial. In addition to the positive effects of the plant supplement generated directly, psychological factors may have enhanced women motivation regarding self-care.

In contrast to our results, the self-care in patients with periodontitis did not improve decisively with intake of JuicePLUS+ in the study of Wiesinger [19].

The nutritional supplement JuicePLUS+ improved psychological, somatic, and urogenital symptoms in the 16 weeks of intake. Looking at analysis group 2, treatment with the supplement resulted in a 47.29% reduction of psychological symptoms, in a 35.55% reduction of somatic symptoms, and in a 44.75% reduction of urogenital symptoms. In the study of Carmignani, treatment with placebo and hormone therapy showed an improvement of the symptoms in approximately the same level. Comparing to the placebo group from the trial of Carmignani, we found no significant difference between the results of both groups, which means there is no significant difference between the effects of placebo and JuicePLUS+ on menopausal symptoms. But also compared with the hormone therapy group, there is no better effect of hormones in the treatment of psychologic and urogenital symptoms as well. However, it should be also stated that hormone therapy is more effective in relieving somatic symptoms as well as total MRS score compared to the results obtained for JuicePLUS+. This leads to the conclusion that JuicePLUS+ is an effective and meaningful alternative for women with contraindication to or adverse effects under hormone therapy and desire of nonhormonal therapy.

Despite conflicting study results, treatment with plant concentrate seems to be effective in improving menopausal symptoms.

### 4.5. Explanation of the Results

The effects of the plant concentrate are not exactly explored in the whole context. In fact we know the 30 sorts of fruits, vegetables, and berries, but the precise composition of JuicePLUS+ and the individual constituents of the plants are not known yet. There are to date 29 published studies on the effect of JuicePLUS+ demonstrating numerous positive effects on the organism. Some studies report a distinctive antioxidant and anti-inflammatory effect [15, 16]. Samman et al. were able to show a reduction in homocysteine as a marker of oxidative stress after taking JuicePLUS+ [13]. Since inflammation might be in relationship with menopausal symptoms, this action of the plant concentrate would be a possible route to explain the reduction of somatic symptoms. Individual factors such as lifestyle variables (smoking, activity level, and BMI), poor nutritional status, and general illness might act as a trigger of increased antioxidants, which themselves are linked with somatic symptoms. Therefore the plant concentrate can replenish metabolic deficiencies, reduce oxidative stress, support the immune system, and improve menopausal symptoms.

Another explanation of the increased feeling of well-being can be the more favorable provision of nutrients. Trials were able to show a significant reduction in the homocysteine level. Elevated amount of homocysteine is associated with development of depressive mood. Nutrients such as folic acid, magnesium, or B-vitamins play an important role in the production of neurotransmitters. A deficiency of neurotransmitters might result in the development of psychologic symptoms like depressive mood [36]. Another trial about the effect of JuicePLUS+ verifies a positive influence of the plant substances on the extent of treatment resistance of chronic periodontitis and a positive sense of well-being [19].

### 4.6. Constructive Suggestions

Some critics may question the duration of nutritional supplement use and think it was too short to cause a clinical response. Just like most authors, we observed the most significant effect on the improvement of menopausal symptoms within the initial 8-week period of supplement intake. Therefore the period of 16 weeks was satisfactory to induce effects on menopausal symptoms [1, 37].

A possible limitation of this study is the small number of participants. Further studies with larger number of patients should be undertaken concerning the effect of JuicePLUS+ in the extent of climacteric symptoms.

## 5. Conclusion

This study shows that daily dietary supplementation with plant substances may constitute an effective alternative therapy to conventional alternative medicine for treatment of psychological symptoms and the somatic symptoms hot flashes and sleeping disturbances. It is not able to improve vaginal dryness and proliferation behaviour of vaginal mucosa. Since many women with climacteric symptoms choose not to undergo hormone therapy, the effect of JuicePLUS+ may be useful to them.

We used the nutritional supplement JuicePLUS+ for achieving a comparable intake of herbal substances in the study. Herbal medicine should not be confused with dietary supplements. Herbal medicine involves the use of plants for medical therapeutic purposes. We know the active ingredients and individual chemicals from most medical plants and understand how they work in the body. In contrast to that, there is a need for further studies to make clear what ingredients are responsible for the observed effects of JuicePLUS+.

## Figures and Tables

**Table 1:** Characteristics of the participants according to analysis groups 1 and 2.

Characteristics	Analysis group 1, *n* = 28	Analysis group 2, *n* = 13
Mean age (years ± SD)	54.86 ± 5.18	55.54 ± 4.89
Age at menopause (years ± SD ) Median (*Q*1–*Q*3)	48.76 ± 5.36 (46.00–52.00)	49.92 ± 2.84 (49.00–51.00)
Time since menopause (years ± SD) Median (*Q*1–*Q*3)	6.76 ± 7.73 (1–11)	5.62 ± 4.35 (3–6)
Time since first symptoms (years ± SD) Median (*Q*1–*Q*3)	2.43 ± 4.52 (0–2.00)	3.08 ± 5.24 (0–2.00)
Body mass index (kg/m^2^) Median (*Q*1–*Q*3)	25.19 (21.20–27.00)	24.83 (21.20–26.60)
Systolic blood pressure (mmHg) Median (*Q*1–*Q*3)	126.61 (120.00–140.00)	124.62 (120.00–130.00)
Diastolic blood pressure (mmHg) Median (*Q*1–*Q*3)	78.75 (70.00–85.00)	77.31 (70.00–80.00)
Baseline total MRS (±SD)^a^	17.86 ± 6.16	17.54 ± 5.39
Baseline somatic symptoms, mean (±SD)^a^	6.82 ± 3.06	6.92 ± 3.48
Baseline psychologic symptoms, mean (±SD)^a^	7.61 ± 3.27	7.00 ± 2.77
Baseline urogenital symptoms, mean (±SD)^a^	3.43 ± 2.63	3.62 ± 2.29

*Q*1, first quartile; *Q*3, third quartile; SD, standard deviation.

^a^Calculated using Menopause Rating Scale.

**Table 2:** MRS subgroups, absolute differences (visit 3 − visit 1), analysis group 1, *n* = 28.

	Subjects	Median	Mean ± SD	IQR
Difference in psychological symptoms	28	−2.00	−3.43 ± 3.04	3.5
Difference in somatic symptoms	28	−2.00	−2.75 ± 3.19	3.5
Difference in urogenital symptoms	28	−1.00	−1.68 ± 2.42	2.0
Summary of differences	28	−7.00	−7.86 ± 5.45	4.5

This table reflects the differences in MRS score between the subgroups at visit 3 and baseline.

SD, standard deviation; IQR, interquartile range.

**Table 3:** Proliferation behaviour in categories, analysis group 1, *n* = 28.

Proliferation behaviour				
Proliferation behaviour baseline	Proliferation behaviour visit 3			
	1	2	3	Total
Frequency row percent				
1, 1-2, 2-1^a^	6	3	0	9
	66.67	33.33	0.00	

2, 2-3, 3-2^b^	1	0	1	2
	50.00	0.00	50.00	

3, 3-4, 4-3, 4^c^	0	0	17	17
	0.00	0.00	100.00	

Total	7	3	18	28

This table shows the change in proliferation behaviour in categories. There appeared to be no statistically important changes for the proliferation behaviour from baseline to visit 3.

^a^Proliferation behaviours 1, 1-2, and 2-1: parabasal calls, small intermediate cells.

^b^Proliferation behaviours 2, 2-3, and 3-2: small and large intermediate cells.

^c^Proliferation behaviours 3, 3-4, 4-3, and 4: large intermediate cells, superficial cells.

^a,b,c^Schmitt's proliferation value [21].

**Table 4:** Comparing with placebo group and hormone therapy group.

Menopause Rating Scale	Analysis group 2, *n* = 13 Difference in mean, 95% CI	Analysis group Carmignani et al. 2010 Placebo group Difference in mean	Analysis group Carmignani et al. 2010 Hormone therapy group Difference in mean
Psychological symptoms	−3.31, * *[−4.87; −1.75]	−2.5	−3.4
Somatic symptoms	−2.46, [−4.27; −0.65]	−2.9	−5.3
Urogenital symptoms	−1.62, * *[−2.55; −0.69]	−0.7	−2.4
Overall MRS	−7.38, * *[−9.91; −4.85]	−6	−11.1

This table shows the differences in mean value in analysis group 2 from our study and in the placebo group and hormone therapy group of Carmignani et al. [22].

CI, confidence interval.
